# After Diagnosing New-Onset Atrial Fibrillation, Be on the Lookout for Venous Thrombosis and Embolization∗

**DOI:** 10.1016/j.jacadv.2023.100556

**Published:** 2023-08-11

**Authors:** Ralph J. Verdino

**Affiliations:** Perelman School of Medicine, University of Pennsylvania, Longboat Key, Florida, USA

**Keywords:** atrial fibrillation, venous thromboembolism

**Figure:**
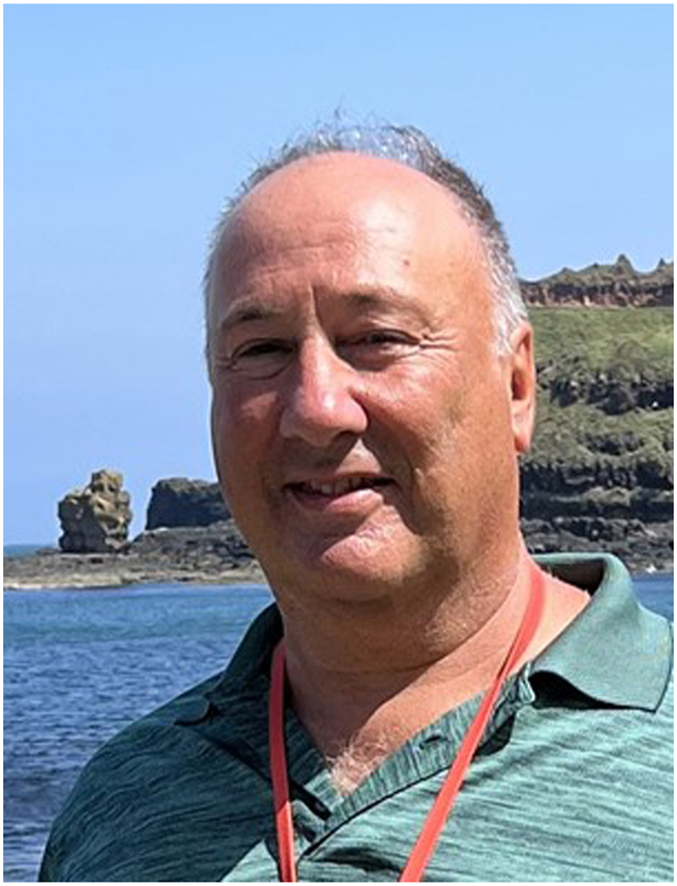

It is widely accepted that stroke is the most serious and feared complication of atrial fibrillation. This fact has long been realized in the medical community and more recently is also well-known with the lay public. Television and other media advertising by the companies that make direct acting anticoagulants and medical devices as well as public service announcements by medical organizations and societies have raised the public awareness of the risk of stroke in patients with atrial fibrillation who are not treated with systemic anticoagulation or a left atrial occlusion device. However, stroke and systemic arterial embolization are not the only thrombotic risks in patients with atrial fibrillation.

In the paper by Pastori et al^1^ in this issue of *JACC: Advances* the risk of venous thromboembolism (VTE) is highlighted. Over the years, the association between atrial fibrillation and venous thrombosis and embolization has been realized since many risk factors associated with atrial fibrillation and arterial thromboembolism are known risk factors for VTE. However, the major finding by the authors is that the risk of VTE is alarmingly high in the early time frame after the diagnosis of atrial fibrillation and seems to significantly wane over time. This is puzzling to me, and raises concern about the best way to decrease the risk of VTE in our patients with atrial fibrillation.

Included in the study by Pastori et al^1^ are 8 studies that report the timing of the first occurrence of VTE after the initial diagnosis of atrial fibrillation. Over 650,000 patients with atrial fibrillation are analyzed and compared with almost 3.5 million individuals not known to have experienced atrial fibrillation. This meta-analysis concludes that the risk of VTE (pulmonary embolism, and deep venous thrombosis) is 6- to 9-fold higher in the first 6 months after the diagnosis of atrial fibrillation as compared to those not found to have atrial fibrillation. It further provides evidence that the risk of VTE after AF diagnosis significantly decreases over time, dropping to a HR with CIs that include 1.0 in the combined analysis of the studies with long-follow-up over 6 months.

The authors postulate that the reasons for the highest risk of VTE early after the diagnosis of atrial fibrillation may be multifactorial but favor the low quality of anticoagulation in the first 90 days in patients treated with vitamin K antagonists. Unfortunately, data regarding anticoagulation treatment were available only for 4 of the 8 studies. The percentage of patients receiving anticoagulation was widely variable and dismally low, ranging from 23.5% to 41.2% of patients. The authors base their theory on that fact that the time period early after atrial fibrillation diagnosis is also the one with the highest risk of thromboembolic stroke. If the risk factors for atrial fibrillation and venous thrombosis and embolisms are so similar that they occur in the same groups of patients, then the risk of experiencing a venous thrombotic event should be consistent for patients with atrial fibrillation or even worsen over time (as most risk factors worsen as we age). This would also be true if atrial fibrillation increased the thrombotic milieu—a theory that appears physiologically sound. After all, the stasis of blood in the left atrium (especially in the left atrial appendage) which leads to stroke and systemic embolization, likely occurs in the right atrium (and the right atrial appendage) and leads to pulmonary emboli (one of the end points in this meta-analysis). However, if there were effective treatments to prevent thrombosis, such as anticoagulants, then the risk should diminish over time as the treatment is initiated.

If the poor quality of anticoagulation is the reason for the high incidence of VTE in the first 6 months after atrial fibrillation diagnosis, what does it say about medical practice? Several of the studies included in the meta-analysis occurred when vitamin K antagonists were the predominant anticoagulant used to treat atrial fibrillation, and it is difficult to regulate early on. But does it really take 6 months? And, do we really get it so right afterward that the increased incidence of VTE essentially disappears? Do the low rates of anticoagulation seen in several studies included in this meta-analysis really improve over time such that the risk of VTE seen in patients with atrial fibrillation approaches those without atrial fibrillation?

Several of the studies in this meta-analysis did use the direct acting anticoagulants which are much easier to use, and give a quite consistent effect almost from initiation of the medication. Could the answer to this conundrum be patient noncompliance early on? Or is it physician reluctance to prescribe anticoagulants for patients diagnosed with atrial fibrillation? These are both worrisome possibilities, and if true, both require significant attention of the medical community. But again, why the improvement seen at 6 months?

Another theory suggested by the authors is the fact that patients with atrial fibrillation are more frequently seen by physicians after a new diagnosis and may have their other medical issues more likely to be diagnosed. This is true as the diagnosis of cancers seem to increase after a diagnosis of atrial fibrillation, and probably is not causally related. Certainly, bleeding caused by anticoagulation initiation may bring an occult cancer to a patient’s and physician’s attention, but the more frequent follow-up probably plays a role. This may contribute to the findings in the study by Pastori et al,^1^ but are not most people with VTE somewhat symptomatic, and seek medical attention themselves? And even if a small percentage of patients are on adequate doses of anticoagulation, should not the incidence of VTE decrease in patients with atrial fibrillation?

You might think that a paper that includes analysis of over 4 million patients would lead to definitive conclusions and recommendations for patient care. I think the authors did an excellent job in shining a light on major health issues that is likely to worsen over time. As populations age, atrial fibrillation becomes ever more common, and so may issues with VTE. Unfortunately, this manuscript does not solve the problems it so successfully brings to our attention. It is our job as medical practitioners and researchers to connect the dots and lessen the incidence of VTE early after the diagnosis of new onset atrial fibrillation so well highlighted by this meta-analysis. This manuscript does an excellent job in identifying the major medical problem of the high rate of VTE early after the diagnosis of atrial fibrillation, now we need to take the next steps in solving this problem.

## Funding support and author disclosures

The author has reported that he has no relationships relevant to the contents of this paper to disclose.
